# Cryptococcal meningoradiculitis presenting with acute flaccid paralysis

**DOI:** 10.1590/0037-8682-0192-2021

**Published:** 2021-04-28

**Authors:** Chee Yik Chang

**Affiliations:** 1 Hospital Sultanah Aminah, Department of General Medicine, Johor, Malaysia.

A 38-year-old man with newly diagnosed advanced acquired immunodeficiency syndrome (AIDS) (CD4 count = 17 cells/mm^3^) presented with bilateral lower limb weakness associated with bowel and urinary incontinence. Neurological examination revealed hypotonia, areflexia, paralysis of bilateral lower limbs (power = 0/5), and sensory loss below the level of T12. Magnetic resonance imaging (MRI) showed spinal meningeal enhancement surrounding the conus medullaris from the upper T12 level with enhancement of the cauda equina nerve roots (Figure 1). Lumbar puncture revealed elevated opening pressure (40 cm H_2_O) and colorless cerebrospinal fluid (CSF) with normal glucose and protein levels. Cryptococcal antigen was present in high titer in the CSF (1:1024), and CSF culture revealed *Cryptococcus neoformans*. The nerve conduction study demonstrated mixed sensory and motor axonal neuropathy of the bilateral lower limbs, sparing the upper limbs. The clinical, electrophysiological, and radiological findings were suggestive of cryptococcal meningoradiculitis. Intravenous amphotericin B and flucytosine were started. However, the patient did not achieve neurologic improvement and eventually succumbed to nosocomial infection. 

**FIGURE 1:**
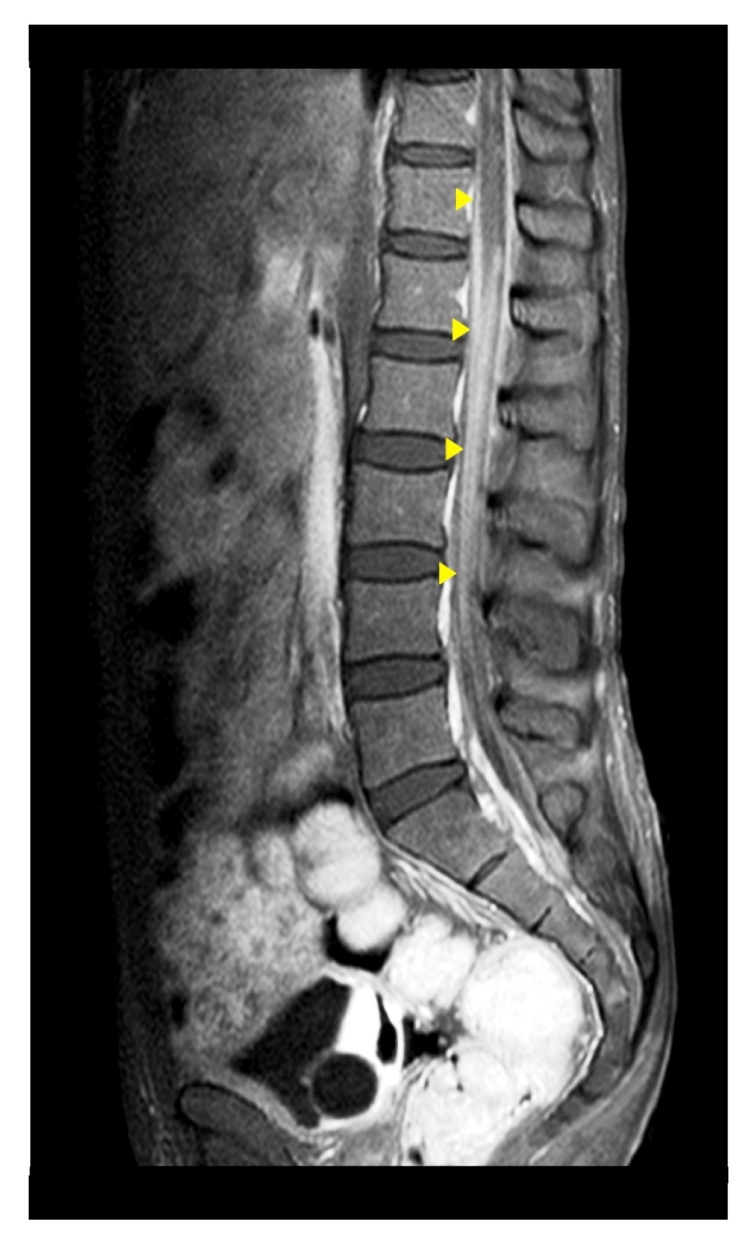
MRI revealing spinal meningeal enhancement surrounding the conus medullaris from the upper T12 level downwards (arrow) with an enhancement of the cauda equina nerve roots.

Cryptococcal meningitis is an important opportunistic infection caused by the encapsulated yeast *C. neoformans* ^1^. Neurological manifestations of cryptococcal disease include meningitis, myelitis, encephalitis, and cryptococcoma^2^. Meningoradiculitis is a rare form of involvement in cryptococcal infection, involving the meninges and nerve roots. Cryptococcal meningoradiculitis can manifest as immune reconstitution inflammatory syndrome in patients with AIDS following initiation of antiretroviral therapy^3^. Cryptococcal meningoradiculitis is a rare condition that should be suspected in HIV-infected individuals presenting with acute flaccid paralysis.

## References

[1] Bicanic T, Harrison TS (2005). Cryptococcal meningitis. Br Med Bull.

[2] Mitchell DH, Sorrell TC, Allworth AM, Heath CH, McGregor AR, Papanaoum K (1995). Cryptococcal disease of the CNS in immunocompetent hosts: influence of cryptococcal variety on clinical manifestations and outcome. Clin Infect Dis.

[3] Jongwutiwes U, Malathum K, Sungkanuparph S (2007). Cryptococcal meningoradiculitis: an atypical presentation after initiation of antiretroviral therapy. J Med Assoc Thai.

